# Using root economics traits to predict biotic plant soil-feedbacks

**DOI:** 10.1007/s11104-023-05948-1

**Published:** 2023-03-02

**Authors:** Gemma Rutten, Eric Allan

**Affiliations:** Institute of Plant Sciences and Oeschger Centre for Climate Change Research, University of Bern, Altenbergrain 21, 3013 Bern, Switzerland

**Keywords:** Root economics, Root traits, Mutualists, Pathogens, Plant-soil feedbacks, Soil communities

## Abstract

Plant-soil feedbacks have been recognised as playing a key role in a range of ecological processes, including succession, invasion, species coexistence and population dynamics. However, there is substantial variation between species in the strength of plant-soil feedbacks and predicting this variation remains challenging. Here, we propose an original concept to predict the outcome of plant-soil feedbacks. We hypothesize that plants with different combinations of root traits culture different proportions of pathogens and mutualists in their soils and that this contributes to differences in performance between home soils (cultured by conspecifics) versus away soils (cultured by heterospecifics). We use the recently described root economics space, which identifies two gradients in root traits. A conservation gradient distinguishes fast vs. slow species, and from growth defence theory we predict that these species culture different amounts of pathogens in their soils. A collaboration gradient distinguishes species that associate with mycorrhizae to outsource soil nutrient acquisition vs. those which use a “do it yourself” strategy and capture nutrients without relying strongly on mycorrhizae. We provide a framework, which predicts that the strength and direction of the biotic feedback between a pair of species is determined by the dissimilarity between them along each axis of the root economics space. We then use data from two case studies to show how to apply the framework, by analysing the response of plant-soil feedbacks to measures of distance and position along each axis and find some support for our predictions. Finally, we highlight further areas where our framework could be developed and propose study designs that would help to fill current research gaps.

**Supplementary Information:**

The online version contains supplementary material available at 10.1007/s11104-023-05948-1.

## Introduction

Interactions between plants and soil biota that affect the subsequent growth of conspecific or heterospecific plants are referred to as plant soil feedbacks (Bever 2003; Van der Putten et al. 2013). Such biotic plant soil feedbacks (PSFs) are ubiquitous and have wide-ranging ecological effects (Van der Putten et al. 2013). For example, PSFs can affect plant population dynamics (Bennett et al. 2017; Crawford et al. 2019), plant abundance (Mangan et al. 2010; Rutten et al. 2016; Reinhart et al. 2021), diversity (Teste et al. 2017) and biodiversity-ecosystem functioning relationships (van der Heijden et al. 2008; Maron et al. 2011; Schnitzer et al. 2011; Mommer et al. 2018; Forero et al. 2021). However, it remains hard to forecast the outcome of plant soil feedbacks, in part due to an incomplete understanding of the mechanisms underlying PSFs.

In the last years, several quantitative reviews have improved our understanding of PSFs. They show that negative plant soil feedbacks dominate, which suggests an important role for pathogens in determining PSFs (Kulmatiski et al. 2008; Lekberg et al. 2018; Crawford et al. 2019; Reinhart et al. 2021). However, the strength and direction of PSFs can vary substantially and factors such as plant functional group and growth form, plant native status, evolutionary relatedness, plant abundance and local environmental conditions can explain the outcome of PSFs (Kulmatiski et al. 2008; Lekberg et al. 2018; Crawford et al. 2019; Reinhart et al. 2021). Several reviews have evaluated different approaches to testing plant-soil feedbacks and have pinpointed knowledge gaps in the field. However, large parts of the variation in PSFs remained unexplained in previous meta-analyses (Lekberg et al. 2018; Crawford et al. 2019), suggesting that we need to consider additional predictors of plant-soil feedback strength.

Box 1. Measuring PSFs

**Table:** 

**Fig. 1:**
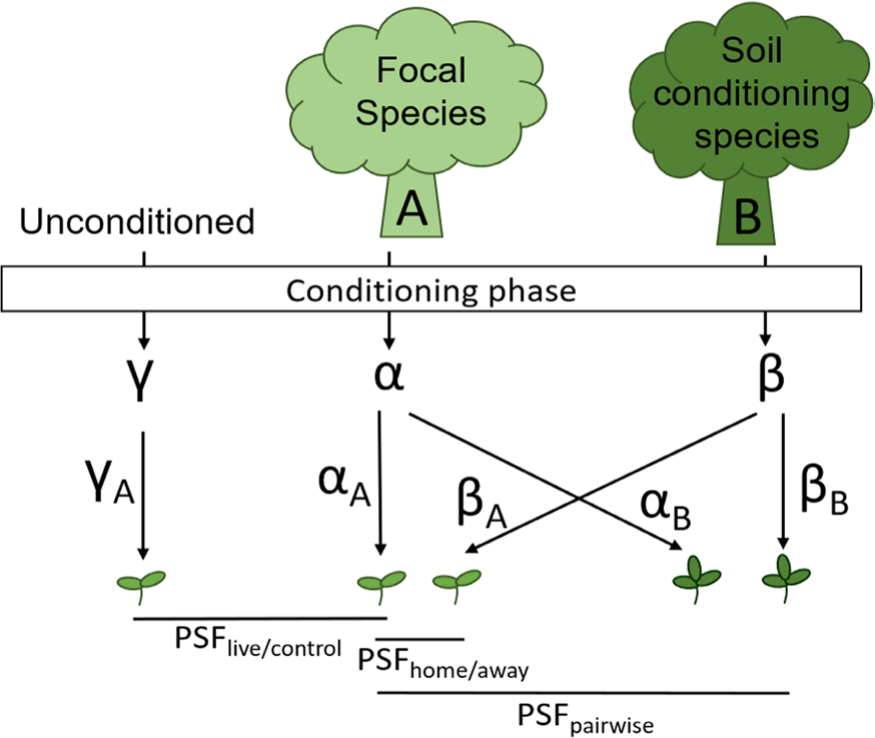
PSF metrics have in common that they compare the growth of a focal species (**A**) on soil conditioned by the same species (α) versus on a control soil. The control can be unconditioned soil (γ) or soil conditioned by another species (β). Pairwise feedbacks reciprocally compare the performance of focal species (**A**) and soil conditioning species (**B**) on their respective soils α and β, where α_A_ is A’s performance in conspecific soil, α_B_ is B’s performance in heterospecific soil, β_A_ is A’s performance in heterospecific soil, β_B_ is B’s performance in conspecific soil. Consequently, feedback can be measured as PSF_home/away_ (α_A_ – β_A_), PSF_pairwise_ (α_A_—α_B_—β_A_ + β_B_) or PSF_live/control_ (α_A_—γ_A_). Note that ‘away’ soils are often pooled across species in a community and ‘control’ soils can be unconditioned, sterilized, fungicide or AMF additions

*Multiple experimental approaches have been proposed to quantify PSFs, each with their own metric *(Fig. 1)*. The most commonly-used approach is to calculate the PSF*_*home/away*_*, which compares plant performance on soil trained by own vs other plant species (Kulmatiski et al. *2008; *Lekberg et al.* 2018*). The advantage of this metric is that it assesses the net effects of the species-specific soil communities. However, the home/away approach cannot assess which soil community component is responsible for the PSF, nor does it directly predict the outcome of competitive interactions. The pairwise feedback approach, PSF*_*pairwise*_*, compares the performance of two plant species growing separately in their respective soils, which corrects for overall differences between soils and relates more closely to the capacity of the pair of plant species to coexist or not (Bever et al.* 1997; *Crawford et al.* 2019).
*The last approach, PSF*_*live/control*_*, compares plant performance on soil trained by a particular plant species vs an unconditioned control soil. Variations of this approach include comparing home/sterile, away/sterile, home/unconditioned and home/fungicide, where the effects of specific agents can be isolated (Petermann et al.* 2008; *MacDougall et al.* 2011; *Bagchi et al.* 2014; *Rutten et al.* 2016*; Lekberg et al.* 2018*). This metric isolates the effects of one soil community, where negative feedbacks suggest an accumulation of pathogens and positive PSF an accumulation of mutualists. All these three approaches mostly use small amounts of inoculum added to a common background soil, to isolate the biotic feedback and reduce the effects of other drivers, such as differences in nutrient availability or nutrient flush after sterilization (Brinkman et al.* 2010*). The proportion of inoculum added, ranges from 0.8–100% across studies, but this did not consistently affect strength of PSFs in a recent meta-analysis (Crawford et al.* 2019*).*
*With this, each of the three commonly-used plant soil feedback metrics answers a slightly different question. PSF*_*home/away*_* evaluates the effects of the specialized soil community cultured by a particular plant species by comparing its effect with a soil community cultured by one or more other plant species, where only generalist taxa should affect plant growth. PSF*_*pairwise*_* evaluates how changes in soil communities conditioned by different plant species affect interactions between them. Finally, PSF*_*live/control*_* evaluates the net effects of a soil community including both its specialized and generalist components, where the "control" soils may be unconditioned, sterilized, fungicide or AMF soils. However, whilst almost all PSF studies test the growth of species on their home soils (α) the control soils that are used for comparison vary substantially, e.g., they can be unconditioned or sterilised or treated soil; γ or soil conditioned by one or more other species; β. Therefore, it is important to carefully consider the control soils used as these determine which conclusion can be drawn. A combination of approaches likely leads to the best understanding but is also most labour intensive.*

## Ecological theory and agents of plant soil feedback

Plant soil feedbacks have been explained by various agents and ecological theories. Most ecological theories assume pathogens are the main agents of PSF because studies predominantly find negative PSFs (Table 1), independent of the metric used (Box 1). Such negative feedbacks are thought to be caused by host-specific pathogens, in line with the Janzen-Connell hypothesis (Janzen 1970; Connell 1971), which predicts that host-specific enemies prevent any one plant species from outcompeting the others, by causing negative intraspecific density dependence (Petermann et al. 2008; Mangan et al. 2010; Bever et al. 2015). The Janzen-Connell hypothesis therefore provides a promising mechanism for the stabilisation of species coexistence (tested by PSF_pairwise_). Mutualists in contrast are expected to drive postive PSF_live/control_ and if the mutualists are specialised they should typically cause positive PSF_home/away_ and PSF_pairwise._ However, plant soil feedback experiments compare two complete soil communities, which means that, a negative PSF_home/away_ metric can also result from a net antagonistic effect on home soil or a net mutualistic effect on away soil or both (Bever et al. 1997; McCarthy-Neumann and Kobe 2010a; MacDougall et al. 2011; Rutten et al. 2016). To separate these effects, an additional control soil can be used, for example unconditioned or sterilized soil (Box 1). This way negative feedbacks (PSF_live/control_) indicate a net antagonistic effect that is very likely brought about by pathogens. Ecological concepts related to mutualistic agents of PSFs often focus on resource availability and local adaptation (Table 1). The idea is that plants seek to optimize the capture of the most limiting resource and invest their photosynthate into symbionts under suboptimal conditions (optimal allocation model), to achieve this, soil communities might be adapted to the local conditions to optimize nutrient uptake and cycling, resulting in positive PSF_home/away_ (Johnson et al. 2010; Revillini et al. 2016). Such local adaptation has also been found in decomposer communities (Home-field advantage), where decomposition of recalcitrant litter accelerated in home soils (Hunt et al. 1988; Ayres et al. 2009; Veen et al. 2015). Moreover, accelerated turnover of labile litter has been predicted to result in negative PSF_live/control_, whereas recalcitrant litter likely results in positive PSF_live/control_ (Semchenko et al. 2022). Therefore, PSFs are likely to be mediated by both pathogenic and mutualistic agents in the soil but it remains challenging to predict which soil component is important under which conditions.

**Table 1:**
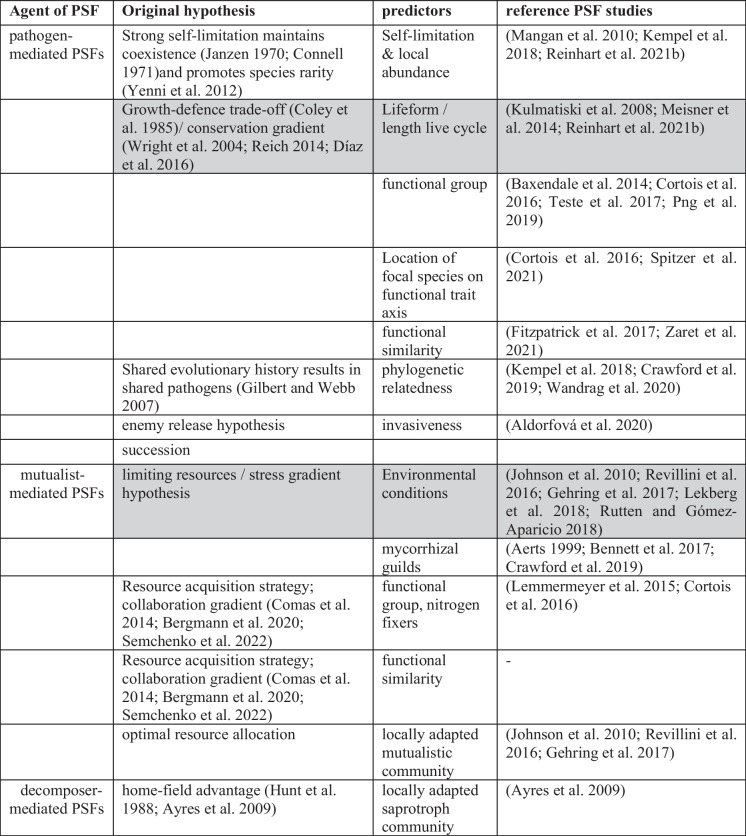
Overview of PSFs in literature. Plant-soil feedbacks have been explained by different agents and theories, using various predictors within (not shaded) and across plant communities (shaded). The majority of the studies refer to pathogen mediated plant soil feedbacks while fewer theories and studies explain PSFs mediated by mutualists and decomposers

## Predictors of plant soil feedbacks

Previous attempts to predict the outcome of plant soil feedbacks often focus on eco-evolutionary factors such as growth form, plant abundance, functional group and life history strategy to explain the outcome of PSFs. For example, grassland species, particularly grasses and annual herbs, generally show stronger negative feedbacks than perennials (Kulmatiski et al. 2008; Meisner et al. 2014) and rare plant species often experience stronger negative PSFs than locally abundant species (Klironomos 2002; Mangan et al. 2010; Rutten et al. 2016; Kempel et al. 2018; Reinhart et al. 2021). Furthermore, the outcome of PSFs between a pair of species might depend on their phylogenetic relatedness, as closely-related plant species might share more pathogens than distantly related plants. By assessing PSF_live/control_ on soils conditioned by different plant species, studies found evidence for an accumulation of host-specific pathogens (McCarthy-Neumann and Kobe 2010a, b; MacDougall et al. 2011; Cortois et al. 2016; Rutten et al. 2016), and showed that the effects of these host-specific pathogens become more neutral with increasing phylogenetic distance between the focal species (home) and the species that conditioned the away soil (Kempel et al. 2018). In contrast, feedbacks that directly compare the competitive abilities of a pair of plant species (PSF_pairwise_) expect, and find, neutral feedbacks for close relatives, as they accumulate similar soil biota and respond similarly to each other’s soil biota (Crawford et al. 2019; Wandrag et al. 2020). Thus, when evaluating the effects of phylogenetic distance on PSFs it is important to consider the control treatment and the PSF metric.

Finally, functional traits and plant nutrient acquisition strategies have been used to predict the outcome of PSFs. For example, functional traits from the leaf economics spectrum (LES), and the related fast-slow strategies have been proven helpful in predicting PSFs (Baxendale et al. 2014; Cortois et al. 2016; Teste et al. 2017; Png et al. 2019). Focal plant species with fast life histories traits, indicated by high specific leaf area (SLA), N content and specific root length (SRL), suffered most from negative feedbacks (PSF_home/away_), whereas species with more conservative traits, such as high dry matter content or average root diameter, showed more neutral or positive feedbacks (Cortois et al. 2016; Spitzer et al. 2022). So far, most studies use the functional traits of the focal plant species to predict the outcome of PSFs, but in theory, similarity in functional traits could be used in a similar way to phylogenetic similarity to predict the outcome of PSFs between plant species. However, testing for distance effects requires that particular home and away soils are compared and only a few studies have looked at how trait distance affects PSF (Table 1). One study found negative litter feedbacks for ten Asteraceae species, but neither phylogenetic distance nor functional distance predicted the strength of these negative feedbacks (Zaret et al. 2021). However, they calculated multivariate functional distances based on multiple traits, which might have obscured opposing effects of individual traits. Another study by Fitzpatrick and colleagues (2017) independently assessed eight functional traits of nine focal species on soils conditioned by 49 co-occurring plant species ranging in phylogenetic distance (PSF_home/away_). They showed that trait differences between soil-conditioning and focal plants may lead to both positive and negative soil feedbacks (Fitzpatrick et al. 2017). Hence plant traits, particularly traits related to growth-defence trade-offs, can help predict the outcome of PSFs but future studies should move from using focal species ‘home’ traits alone to considering the distance between pairs of plant species in order to explain variability in PSFs.

## Links between below ground plant strategies and soil community components

**Fig. 2:**
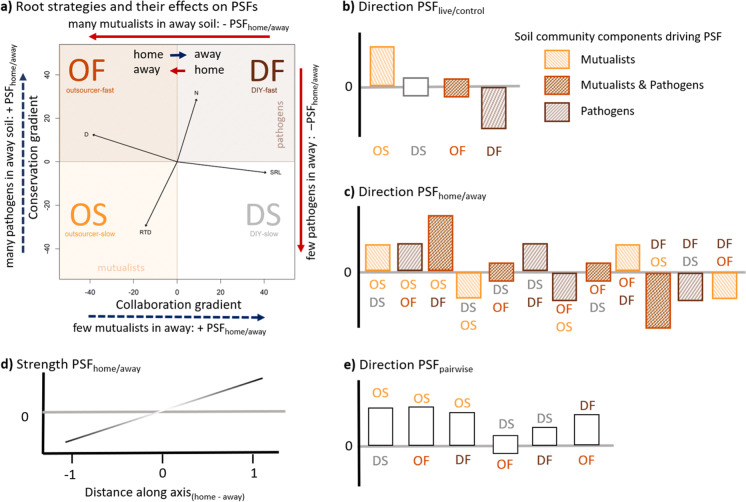
Links between root economics space and plant soil feedbacks. The collaboration axis is expected to link more strongly to mutualistic effects and ‘outsourcer’ species should accumulate mutualists in their home soils (a; yellow shaded area). The conservation axis on the other hand, more strongly links to pathogenic effects and fast species should accumulate pathogens in their home soils (a; brown shaded area). This results in four belowground strategies each with a unique combination of soil pathogens and mutualists (as indicated by the colours in space). Each strategy shows specific feedback, that can be measured using different metrics (Fig. b-e). b) The four PSF_live/contol_ show the effects of the accumulated soil community on home soils compared to sterilized soil. c) There are 12 possible PSF_home/away_ if each of four (home) strategies is compared against the three remaining (away) strategies. The direction of the PSF_home/away_ will depend on which strategies (two letter abbreviations) are compared and the predicted changes in mutualistic (x-axis) and pathogenic (y-axis) communities are indicated by the arrows along the axes (a). d) Additionally, we expect that the strength of the PSFs is determined by the distance between the home species and the away species along the two axes. If the two species compared have similar strategies (low trait distance), then weak PSFs are expected. However, more positive distances between away – home locations along an axis (blue dashed arrows), will result in progressively stronger positive feedback, as plants either lose mutualists in away soil (Fig a; x-axis) or gain pathogens in away soil (Fig a; y-axis), whereas increasing negative distance (red full arrows), will result in progressively stronger negative feedbacks (d) as plants either gain mutualists (a; x-axis) or escape pathogens in the away soil (a; y-axis). Finally, we also predict the outcome of the six pairwise feedbacks between species of different strategies, i.e., where home and away soils are reciprocally compared (e)

Belowground plant trait strategies also form a conservation gradient, where roots either invest in growth (high root nitrogen content) or defence (high tissue density). This is similar to the leaf economics spectrum aboveground (Wright et al. 2004; Reich 2014; Díaz et al. 2016) but is an independent axis of variation (Carmona et al. 2021). Recently, the fungal collaboration gradient was added as another key axis in root economics space (Comas et al. 2014; Kramer-Walter et al. 2016; Bergmann et al. 2020; Weigelt et al. 2021). The collaboration gradient separates roots that invest in attracting symbiotic fungi (with high root diameter) from those that acquire nutrients mostly by themselves (high Specific Root Length), and the gradient holds across ecotypes and mycorrhizal guilds (Comas et al. 2014; Bergmann et al. 2020). This extension of root economics space suggests an extension of belowground strategies, particularly important for the outcome of PSFs (Fig. 2; Semchenko et al. 2022). Besides the well-established growth and defence strategies, on the fast-slow continuum, an additional orthogonal axis suggests trade-off between do-it-yourself (DIY) vs outsourcing in terms of nutrient acquisition (Semchenko et al. 2022). This theoretically results in four distinct belowground strategies, DIY-slow, DIY-fast, outsourcing-slow and outsourcing-fast (Fig. 2). Interestingly, mutualistic fungi scale on the collaboration axis while pathogenic fungi are thought to mainly relate to the conservation axis (Semchenko et al. 2022). Together, these axes result in four distinct plant strategies, each with its own combination of mutualists and pathogens (Fig. 2). Therefore, linking this root economics space to plant soil feedbacks seems a promising way to better predict the direction and strength of feedbacks between pairs of plant species (Semchenko et al. 2022), but a framework specifically predicting feedbacks between pairs of plants with different strategies has not been developed.

## The conceptual framework

Here we present a framework that predicts the strength and direction of feedbacks between co-occurring plants differing in these four belowground strategies (Fig. 2). We expect that plants from the outsourcer-slow strategy (OS), invest in collaborating with mycorrhiza and do not harbour many pathogens because their roots are well defended. DIY-slow species (DS) in contrast do not invest much in mycorrhiza but are still well protected against pathogens, meaning they accumulate few microbes. Outsourcer-fast species (OF) invest in mycorrhiza but harbour many pathogens because these fast-growing species would be expected to invest in root growth rather than defence compounds in their roots. Finally, DIY-fast species (DF) harbour many pathogens but invest little in mycorrhiza. These strategy-specific effects on the soil communities, are therefore expected to result in a net positive effect of the soil community on plants of the outsourcer-slow strategy (because the mutualists increase growth), a net neutral effect of the soil on plants with the DIY-slow (because the plants have little effect on either pathogens or mutualists) and outsourcer-fast strategies (because the effects of pathogens and mutualists balance each other out) and a net negative effect for the DIY-fast strategy (because the pathogens reduce growth). To test these predictions, we can isolate the net effects of the home soil communities on plant growth using the comparison PSF_live/control_ (Fig. 2b). Note that we assume the many agents driving plant-soil feedbacks are microbes but pathogenic nematodes or root feeding insects may also respond to the fast-slow axis and accumulate in soil of fast-growing plant species.

Based on these assumed effects of the strategies on soil pathogens and mutualists we can infer the feedbacks between any pair of strategies. We present the predictions for feedbacks calculated using the metric PSF_home/away_, as it is very commonly employed. If we compare each of the four strategies against the remaining three, we can have a total of 12 possible feedbacks. The predictions for each of the 12 are shown in Fig. 2c. For example, we expect a negative feedback when the growth of an outsourcer-fast species is compared on home soil and away soil cultured by an outsourcer-slow (OF-OS) species. Outsourcer-fast plants will accumulate both mutualists and pathogens in their home soil, while outsourcer-slow plants culture mostly mutualists in their home soil. Outsourcer-fast plants will therefore perform better in outsourcer-slow soil as they escape from the pathogens cultured in their home soil (Fig. 2a; red arrow y-axis), resulting in negative PSF_home/away_ (Fig. 2c). However, the feedback between outsourcer-fast and DIY-fast (OF-DF) strategies is expected to be positive, because the outsourcer-fast plants will lose their mutualists when growing in away soil cultured by DIY-fast plants (Fig. 2a; blue arrow x-axis), and therefore they will perform best in home soil (Fig. 2c). We also expect a neutral feedback between outsourcer-fast and DIY-slow plants (OF-DS) because outsourcer-fast plants will culture both pathogens and mutualists, while DIY-slow plants will not have large effects on the soil community. If we assume that pathogen and mutualist effects are perfectly balanced, we should therefore see no difference between home and away soils. In contrast, we would expect a strong negative feedback between DIY-fast and outsourcer-slow plants, because the DIY-fast plants will lose their pathogens and gain mutualists when grown on outsourcer-slow soil, resulting in much worse performance at home. Throughout we therefore also assume that pathogens and mutualists are generalist and able to attack or benefit plants of all other strategies. We discuss the consequences of specialization of the soil microbes below.

**Fig. 3:**
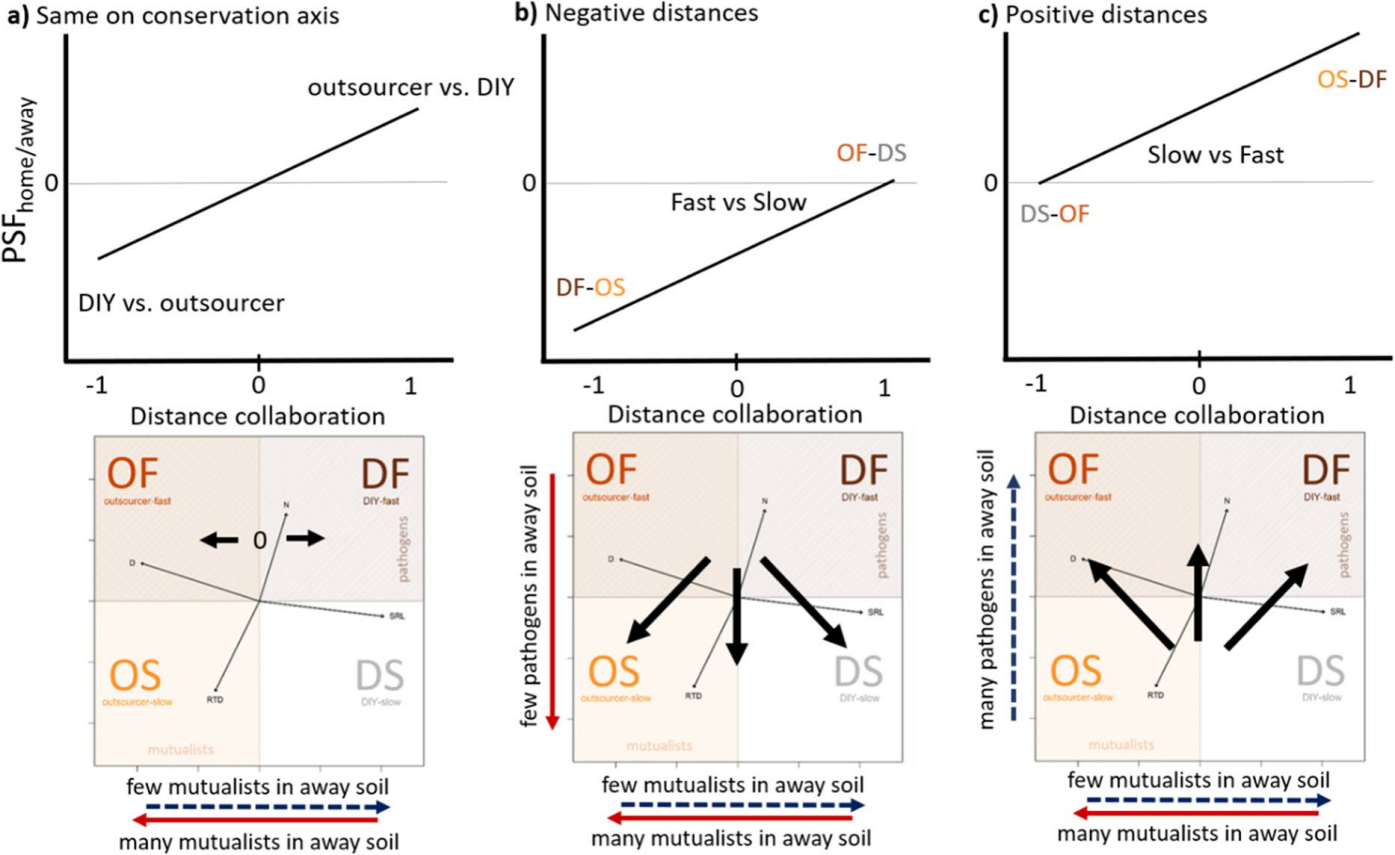
Conceptual figure visualizing how PSFs change along the collaboration and conservation axis of the root economics space. To visualise the expected interaction between the two distances, we show the effect of distance along the collaboration gradient (x-axis) on PSF_home/away_ (y-axis) for three categories of distance along the conservation axis (panels a-c). **a** Predicted PSFs when home and away soils vary along the collaboration axis, but not along the conservation axis (i.e., horizontal arrows in root economic space as indicated in the figure below). At 0 distance along the collaboration axis both species are similar in conservation and collaboration strategy. Increasing positive distances along the collaboration axis indicate a comparison of the growth of outsourcer strategy plants on their home soil vs more DIY strategy away soils (horizontal arrow pointing to the right in the figure below). In contrast increasingly negative distances along the collaboration axis indicate the reciprocal feedback where more DIY strategy plants are grown on home vs more outsourcer strategy away soils (horizontal arrow pointing to the left in the figure below). **b** Predicted PSFs when home and away species also differ along the conservation axis from fast to slow (moving down in root economic space). The 0 point along the collaboration axis indicates the overall feedback between fast and slow species (vertical down pointing arrow in the figure below) and negative distances along the collaboration axis indicate feedbacks between more DIY-fast and more outsourcing-slow species (diagonal arrow from top right to bottom left). Positive distances along the collaboration axis indicate comparisons between Outsourcing-fast and DIY-slow species (diagonal arrow from top left to bottom right). c) Predicted PSFs when home and away species differ along the conservation axis from slow to fast. The 0 point along the collaboration axis indicates the overall feedback between slow and fast species (upward pointing vertical arrow). Negative distances along the collaboration axis indicate feedbacks between DIY-slow and outsourcer-fast species (diagonal arrow from bottom right to top left). Positive distances along the collaboration axis indicate feedbacks between outsourcer-fast and DIY-slow species (diagonal arrow from bottom left to top right). See the text for explanation of how we expect the PSFs to vary with the distances

Although it is interesting to compare feedbacks between species in the corners of the functional space, most species will have somewhat intermediate strategies. We therefore suggest using continuous measures of distance between home and away soils along each axis (Fig. 3). We generally expect that the further apart two species are along either axis, the larger the magnitude of the PSF_home/away_ (Fig. 2d). We calculate functional distances along the phylogenetically corrected PCA1 and PCA2, as each axis is defined by two core traits and therefore, although using the traits themselves would be more generalizable, it would require four distances, resulting in much more complex models. Although the PC-axes produced in studies with different species pools will differ, and therefore species might be placed in different quadrants in different studies, this is not necessarily a problem for our framework because it uses distances between species as the predictor, not a categorical classification into strategies. We define the functional distance as away minus home, so that when a home species at a low point on the axis is compared with an away species at a higher point, this always results in a positive distance (blue dashed arrows, Fig. 2a). When the home species has a higher point on the axis than the away species, this results in a negative distance (red full arrows, Fig. 2a). Positive distances are expected to result in more positive feedbacks (blue dashed arrows, Fig. 2a). This is because growing in soil cultured by a species higher up the up the collaboration axis means losing mutualists in the away soils, whereas growing in a soil cultured by a species higher up the conservation axis means gaining pathogens in away soils, in both cases species should perform better on home soil (Fig. 2a). In contrast, negative distances along either axis result in more negative feedbacks because the home and away soils are reversed (red full arrows, Fig. 2a). Note that this assumes the loadings of the traits on the PCA axes that we show in Fig. 2. If the traits had opposite loadings on the axes, then the PCA can be rotated to match the loadings we show here. As we assume that pathogens and mutualists balance each other out, we expect neutral feedbacks when moving diagonally from outsourcer-fast (many mutualists and pathogens) to DIY-slow (few mutualists and pathogens) or in the opposite direction, because species either lose or gain both mutualists and pathogens at the same time (Fig. 2). In contrast we expect the strongest negative feedbacks when moving from DIY-fast to outsourcer-slow and the strongest positive feedbacks when moving in the other direction because species lose pathogens and gain mutualists, or gain pathogens and lose mutualists in the away soil, respectively. We therefore expect an interaction between the distance along the collaboration and the distance along the conservation axis (Fig. 3).

So far, we have assumed that only the distance between two species affects the strength and direction of the PSF_home/away_. This is because we assume that pathogen and mutualist effects balance each other out and that there is a continuous increase in pathogen accumulation as we move from slow to fast along the conservation gradient and continuous increase in mutualists from DIY to outsourcer. However, these assumptions may be too simplistic and we can further test whether the position of a species pair in root economics space affects the feedback between them. For this, we can calculate the midpoint of the species pair along the collaboration axis (using the phylogenetically corrected PCA1) and the orthogonal conservation axis (using the phylogenetically corrected PCA2). We use the midpoints rather than the home soil position because the home soil position will correlate with the distance (if home soil is at the minimum point along the collaboration gradient, then all distances along the collaboration gradient have to be positive).

With this framework, we can assess if the similarity between the root traits of the home and away species along each axis determines the strength and direction of PSFs, and it allows us to test all possible combinations of belowground strategies (Fig. 2, Fig. 3). Moreover, it benefits our mechanistic understanding of the outcome of PSF as we provide hypotheses for the main soil community components driving the feedbacks in each situation.

## Proof of concept using two case studies

We use two case studies to test our framework and investigate if the outcome of PSFs can be explained by linking below-ground plant strategies to their effects on soil communities. The two case studies cover different plant functional groups and tested several species per functional group, all of which occurred together within a community. Petermann et al. (2008) assessed PSFs of grassland species, including grasses, herbs and legumes. Each species was tested against two species from the other functional groups. Bennett et al. (2017) assessed the PSFs of tree species and included trees that associate with arbuscular- and ecto-mycorrhizal fungi. Both studies included a treatment where the corresponding home and away soils were sterilized. This allows us to simultaneously evaluate the net effects of each soil community (PSF_live/control_) and to compare the effects of two different soil communities on growth of one focal plant species, i.e. PSF_home/away_. Both studies also use co-occurring plants, meaning we avoid calculating PSFs between species that would not grow together.

**Fig. 4:**
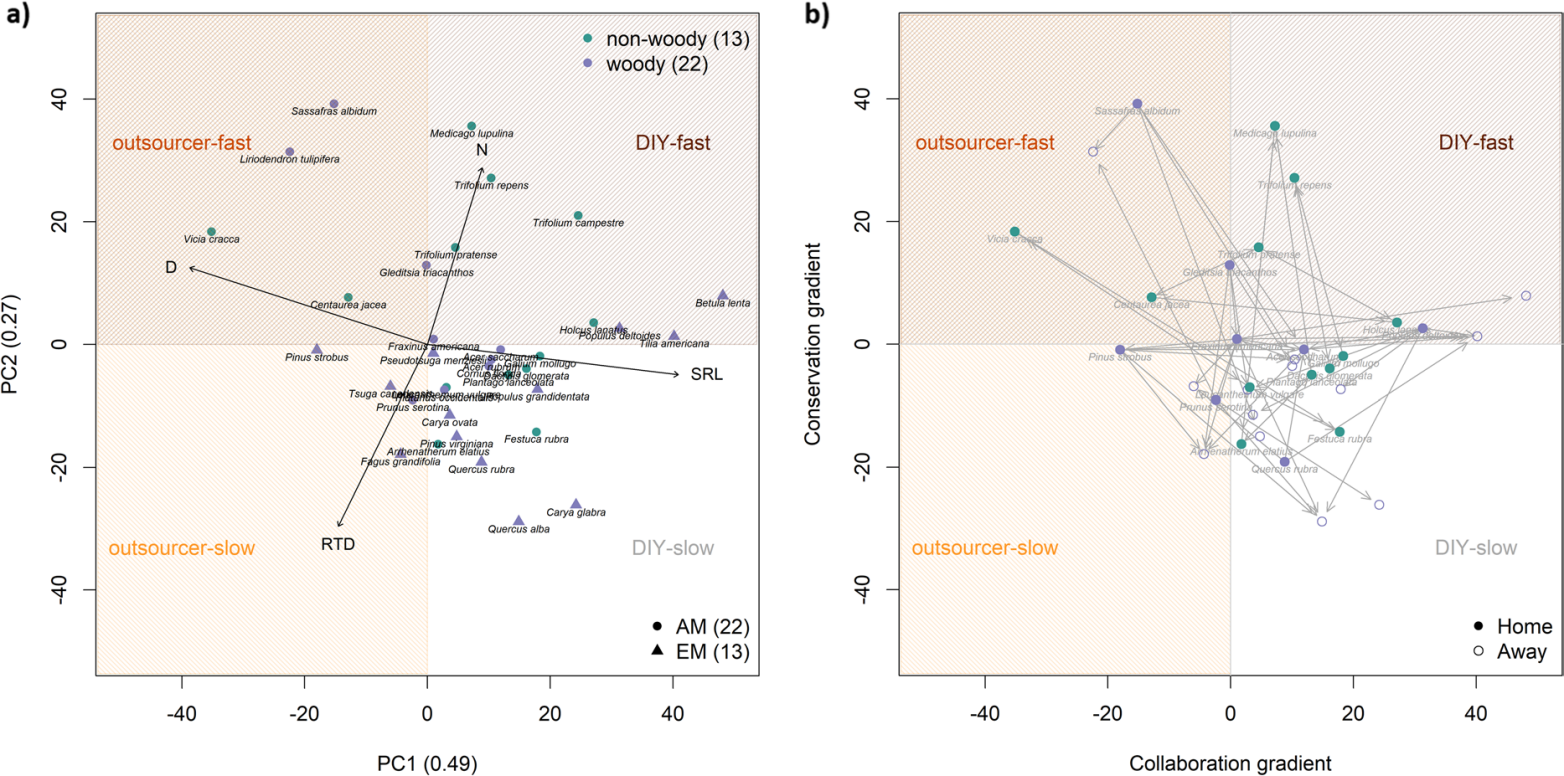
Two PSF case studies in root economics space. **a** Four core root traits (Bergmann et al. 2020; Guerrero-Ramírez et al. 2021): root nitrogen (N), specific root length (SRL), root tissue density (RTD) and root diameter (D), result in a two dimensional root economics space for temperate grassland (Petermann et al. 2008) and forest (Bennett et al. 2017) together. The collaboration axis (49%) ranges from outsourcing (high D) to Do-It-Yourself (high SRL). The conservation axis (27%) ranges from fast (high N) to slow (high RTD). In line with the global root economics space (Bergmann et al. 2020). The collaboration axis is expected to link more strongly to mutualistic effects (yellow shaded area) whereas the conservation axis is expected to link more strongly to pathogenic effects (brown shaded area). Together the case studies included 35 plant species covering all four belowground strategies. **b** The location of focal species (home; filled symbols) and the soil conditioning species (away, open symbols) along the collaboration (x-axis) and conservation (y-axis) gradient, where the difference between the focal species and the soil conditioning species represent the root functional dissimilarity (grey arrows) of the measured PSF_home/away_. The case studies included 61 PSF measures of grassland (green) and forest species (violet). Both case studies included PSF_home/away_ measures and PSF_live/sterile_ measures for each focal species (home; filled symbols). Note that several species were only used as conditioning species (away; open symbols). The data including full species names can be found in the supplementary (Table S1 and S2)

First, we linked the PSFs of our two case studies to root traits collected from the Groot database (Guerrero-Ramírez et al. 2021) to test if root trait similarity can help predict PSFs (Fig. 4a). The database contained full information on Specific root length (SRL), Mean root diameter (D), Root Tissue Density (RTD) and Root Nitrogen concentration (N) for 51% of the case study species (35 of 68 species). These species covered graminoids (4), herbs (4), legumes (5) and trees (22) as well as different mycorrhizal types (Fig. 4a). Following the protocols and criteria for fine roots from Bergmann and colleagues (2020), we selected the fine root traits and pruned a phylogenetic tree (Zanne et al. 2014) for the species used in the two case studies (scripts provided in Rutten and Allan 2023). A phylogenetically informed principal component analysis (PCA) of the root traits revealed a collaboration gradient (49%) and a conservation gradient (27%), in line with the global root economics space (Bergmann et al. 2020). As in the global analysis, both grassland and forest species cover a broad area and the root economics space did not separate species from different habitats (Bergmann et al. 2020). Tree species covered broader areas of the RES than the grassland species, which seems to be particularly true for tree species that associate with ectomycorrhizal (EM) fungi.

Then, we plotted the focal species (home soil) and the soil conditioning species (away soil) in the root economics space using the values of the PCA axis (Fig. 4b). An arrow from the home to the away species represents the dissimilarity of the species pair in root economic space (Fig. 4b). Both case studies included multiple feedback measures per focal species as indicated by multiple arrows going in different directions, connecting home to away species. It is clear that these species combinations were not selected for their dissimilarity in root traits or belowground strategies, and the strategies are represented by different numbers of species and feedbacks. For example, the DIY strategies have most tested feedbacks, whereas outsourcer-slow strategies seem hardly represented in these data. Moreover, soil conditioning species often seem to have the DIY-slow strategy.

**Fig. 5:**
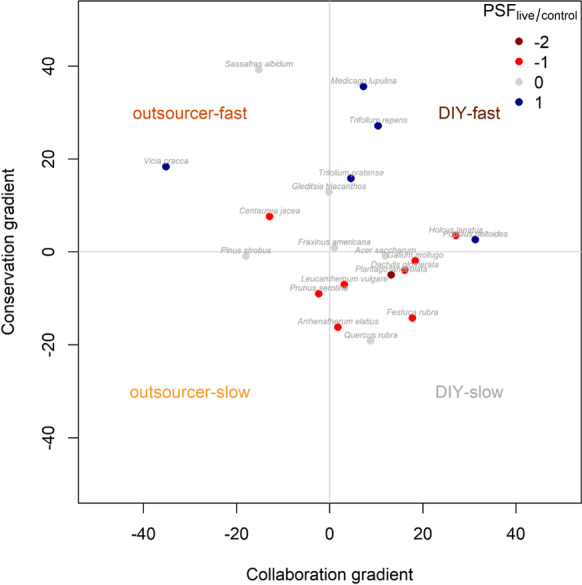
Isolated effects of the home soil communities along the collaboration and conservation axis in root economic space. PSF_live/control_ were categorized into strong negative (dark red), negative (red), neutral (grey) and positive (blue) feedbacks

To test our hypothesis that the net effect of the home soil varies between the four different strategies in root economics space (Fig. 2b), we used a linear model with the PSF_live/sterile_ as the response and the position of the species along the phylogenetically corrected PCA1 (collaboration gradient), the phylogenetically corrected PCA2 (conservation gradient) and their interaction as fixed factors. A significant interaction term provides evidence for the four different strategies having distinct PSFs effects from their home soil communities, which we can visualize by plotting the direction of PSF_live/control_ in root economics space (Fig. 5). We found evidence that the four different strategies have distinct PSFs from their home soils (Table 2a interaction term, F_1,57_ = 5.95*). Contrary to our expectations, the species of our two case studies showed more negative feedbacks at the slow end than at the fast end of the conservation gradient (Table 2; Fig. 5). This suggests that slow growing species accumulate more pathogens than fast growing species, however these results need to be taken with care as the fast species (n = 10) also include legumes (n = 4) that all show positive effects of their own soil microbial communities, possibly due to mutualistic effects of N-fixing rhizobacteria. It would be interesting to design PSF experiments that better cover the two axes of the root economics space with more balanced species selections across the strategies.

**Table 2:** Summary statistics of the effects of root economics space on PSF. **a**) PSF_live/control_ in relation to the location of the focal species on the collaboration and conservation gradient. **b**) PSF_home/away_ in relation to the location and distance measures along both the collaboration and conservation gradient

**a)** PSFl_ive/control_ F_3,57_ = 5.83; R^2^_adj_ = 0.19**	Df	SumSq	MeanSq	F- value	P- value
collaboration gradient (PCA1)	1	0.05	0.05	0.10	0.754
conservation gradient (PCA2)	1	5.66	5.66	11.46	**0.001**
PCA1: PCA2	1	2.93	2.93	5.94	**0.018**
Residuals	57	28.16	0.49		
**b)** PSF_home/away_ F_15,45_ = 4.01; R^2^_adj_ = 0.43***	Df	SumSq	MeanSq	F- value	P- value
collaboration location (coll.mid)	1	4.33	4.33	21.53	** < 0.0001**
conservation location (cons.mid)	1	0.49	0.49	2.43	0.126
collaboration distance (coll.dist)	1	0.03	0.03	0.16	0.694
conservation distance (cons.dist)	1	0.20	0.20	1.00	0.323
coll.mid:cons.mid	1	0.14	0.14	0.69	0.409
coll.mid:coll.dist	1	1.07	1.07	5.31	**0.026**
coll.mid:cons.dist	1	0.29	0.29	1.43	0.237
cons.mid:coll.dist	1	0.05	0.05	0.23	0.631
cons.mid:cons.dist	1	1.21	1.21	6.02	**0.018**
coll.dist:cons.dist	1	0.13	0.13	0.64	0.429
coll.mid:cons.mid:coll.dist	1	0.55	0.55	2.72	0.106
coll.mid:cons.mid:cons.dist	1	0.18	0.18	0.89	0.350
cons.mid:coll.dist:cons.dist	1	0.22	0.22	1.09	0.303
coll.mid:coll.dist:cons.dist	1	0.98	0.98	4.85	**0.033**
coll.mid:cons.mid:coll.dist:cons.dist	1	2.25	2.25	11.18	**0.002**
Residuals	45	9.06	0.20		

In the final step, we linked the distance between the home and away species (Fig. 4b, grey arrows) to the expected changes in mutualistic or pathogenic communities along the collaboration and conservation gradient. We used the signed distance along each axis to indicate the direction of change from home to away soil (as this indicates whether the relative position of the home vs. away soil along each axis; Fig. 2d, Fig. 3). An interaction between the signed distance along each axis provides evidence for the four different strategies having distinct PSFs between different plant species pairs (Fig. 3). Although we hypothesise that only the distances along each axis should affect the PSF, we also tested for effects of the location of the species pair. We calculated the midpoints of the species pair on the collaboration axis (using the mean value of the focal and the soil conditioning species on the phylogenetically corrected PCA1) and the orthogonal conservation axis (using the mean value of the focal and the soil conditioning species on phylogenetically corrected PCA2). If the location on either axis is important, we would see a significant interaction between the midpoints of the two axes.

**Fig. 6:**
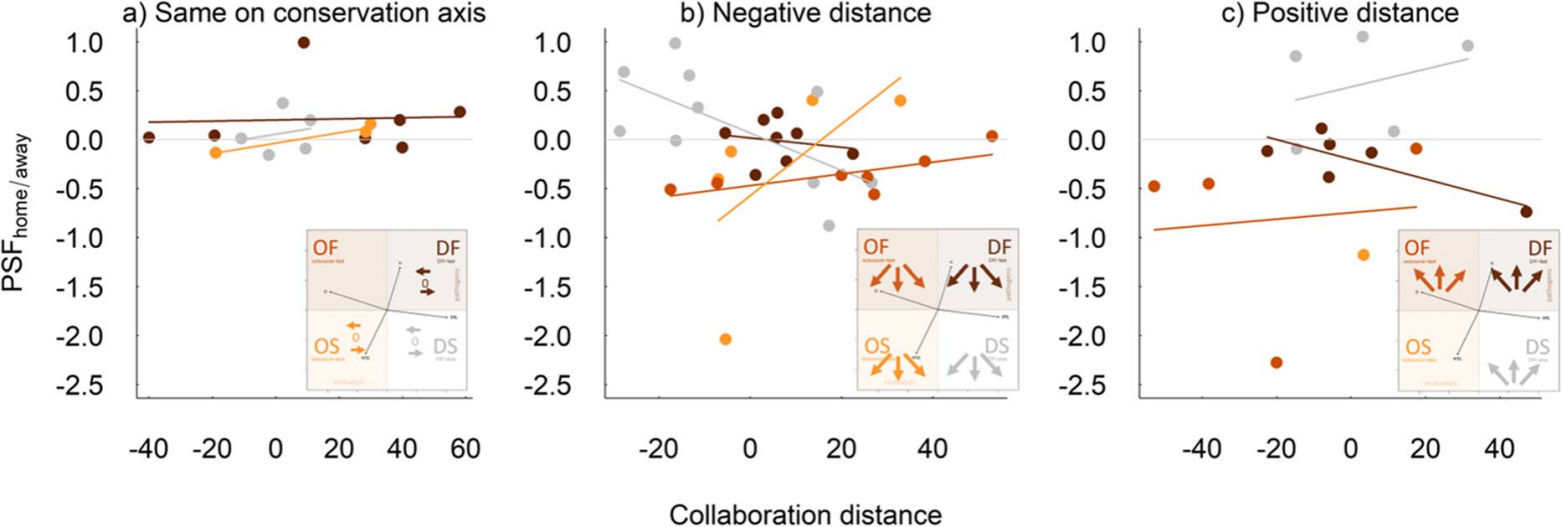
Interactions between the collaboration gradient and the conservation gradient determine the outcome of PSFs in the two case studies used. Effects of distance along the collaboration gradient (x-axis) and distance along the conservation gradient are shown in panels a-c, which correspond to the comparisons shown in Fig. 3. As the mid points along each axis interacted with the distance, we show the effects of the distances for species within each of the four quadrants in root economic space. Inserts visualize the location and direction the species pairs that were tested in the case studies

We found support for our framework combining the collaboration and conservation axis of the root economic space, as our model explained considerable variation in the strength and direction of PSFs (R^2^_Adj_ = 0.43**). This indicates that just using these two overall axes of root trait variation can explain significant variation in PSFs, even in a highly heterogeneous dataset. The significant interaction between conservation and collaboration distance, however, was only found as part of a four-way interaction with conservation and collaboration mid points (Table 2b, Fig. 6). This indicates that, contrary to our expectations, the mid points also help to explain the variation in PSF_home/away_ between species pairs (Table 2b). Though it is challenging to interpret such four-way interactions, we see some evidence for our hypothesis when we plot the interaction between collaboration and conservation distance, for species pairs occupying different positions in the root economic space (Fig. 6). We show the interaction between the distances for mid points falling in each of the four quadrants within root economic space. For example, if the midpoint between the species falls within the outsourcer-slow quadrant, then at least one species must be an outsourcer-slow species and the effect of distance along the conservation gradient would show the comparison between the slowest species and a species with a faster strategy along the conservation gradient (but not necessarily with a species falling in the fast half of the gradient). If both species in the pair have a fairly slow strategy (midpoints fall within the outsourcer-slow and DIY-slow quadrants) then we typically see the predicted interaction between the conservation and collaboration distance. If we look at effects of the collaboration gradient when keeping the conservation gradient constant (Fig. 6a), we see the predicted increase from negative feedbacks when species nearer the DIY end of the gradient culture the home soil and the most outsourcer species cultures the away soil and a positive feedback in the other direction. We also see the predicted relationship when we look at negative distances along the conservation gradient, i.e., a shift from negative feedbacks between more DIY-fast and more outsourcer-slow species to neutral between outsourcer-fast and DIY-slow (Fig. 6 b and 6c). However, the two case studies do not always provide enough data to test all combinations of mid points and distances. In contrast to the cases when both species are from more outsourcer quadrants, the effects of distance are more variable when both species in a pair have a more DIY strategy. The DIY-slow quadrant shows the most variable responses across the two distance axes: it perfectly fits our predictions in the small and positive distances (Fig. 6a and 6c), but shows the opposite effects for negative distances along the conservation gradient. This means that rather than seeing negative feedbacks between more DIY-fast and more outsourcer-slow species, we see positive feedbacks, and between outsourcer-slow and DIY-fast species we see negative feedbacks (Fig. 6). We expected negative feedbacks between DIY-fast and outsourcer-slow species because we expected that DIY-fast species would benefit from the mutualists cultured in outsourcer-slow soils, however, if the DIY species cannot benefit from the mutualistic mycorrhizae (or if the mycorrhizae even act as parasites on them) then we might not see the predicted benefits for DIY species growing in outsourcer soils. When both species belong to the DIY-fast strategy, we never see agreement with our predictions (Fig. 6). The main reason for this might be that all the species showing the most DIY-fast strategy in these datasets are legumes. As legumes culture other bacterial mutualists (rhizobia) they are likely to do well on their own soil and to enjoy positive feedbacks when compared with other species (not the negative feedbacks that we would assume for other DIY-fast species that culture pathogens and few mutualists in their home soils). These results show that our framework can be applied to data to predict variation in PSFs using the root economic space. We urge future studies to consider the relations between the root economics space, using these continuous measures of distance and position in root economic space between species within a pair.

To fully test our framework, we would need to estimate plant-soil feedbacks between a larger set of species, which are more evenly distributed in root economic space, we provide some suggestions on how to design the species selection for such an experiment, below.

## Remaining gaps & future directions

### A thorough test of the framework

In both case studies, we lost about half of the species due to incomplete root trait measures (only 61 of the 127 species pairs had complete root trait entries in the database). It is therefore important for future studies to measure root traits themselves. This might also improve the explanatory power of the root traits as intraspecific variation would be considered. The four root traits that make up the root economic space are amongst the most measured root traits (Guerrero-Ramírez et al. 2021). Though these traits might not be the most direct measures of plant function or plant associations with soil communities, the resulting collaboration and conservation gradient have been shown correlate to physiological traits and mycorrhizal colonisation (Han et al. 2022; Yaffar et al. 2022) and root exudates to a certain degree (Wen et al. 2022). Therefore, we are confident that the traits in the root economics space will prove useful in future studies that assess root strategies and we urge future studies to take up the challenge of measuring belowground traits when performing a PSF study.

It is evident from the case studies that the species used in PSF studies do not completely cover all four belowground strategies and their 12 possible pairwise combinations. This drawback kept us from testing all strategy combinations. In particular, the outsourcer-slow strategy was underrepresented, and generally PSFs comparing fast home soils to slow away soils were tested much more often than the PSFs from slow to fast (Fig. 6). In addition, the DIY-fast strategy was mostly represented by legumes, causing a confounding between the presence of this clade and the traits, which may have led to some discrepancies between predictions and results. Despite these data limitations, we found that our framework, combining the collaboration and conservation axis of the root economic space, explained considerable variation in the strength and direction of PSFs (R^2^_Adj_ = 0.43**), hereby providing first evidence for the importance of belowground functional strategies in affecting the outcome of PSF.

The next step would be to test this concept with a predesigned species pool that better covers all four belowground plant strategies, as well as a broad range in distances along both the collaboration and the conservation axes. Ideally, such an experimental set-up would involve testing the growth of a given species on its own home soil and on several away soils. Testing all pairwise combinations between species is unlikely to be possible however, each species could be grown on soil of a species with a similar strategy, soil of a species distant along the conservation axis, distant along the collaboration axis and distant along both axes. This would generate a wide range of distances to be tested in the model. Such a design would not only test the proposed framework more rigorously, but it could also help to move the PSF-field to more functional approaches by comparing pairs of strategies instead of species.

Our framework currently assumes that the net effects of gaining mutualists and losing pathogens are similar. This means that PSFs between DIY-slow and outsourcing-fast strategies would result in neutral PSFs as plants gain mutualists on away soils and losing pathogens on home soils at the same time (). However, this may be too simplistic, and we might expect negative or positive feedbacks if either pathogens or mutualists have stronger effects. In addition, we assumed that feedbacks are symmetrical along each axis, e.g., that outsourcers suffer from losing their mutualists when grown in DIY cultured soil to the same extent that DIY species benefit from gaining mutualists in outsourcer soil. However, if DIY species do not actually benefit from the mutualists, then this might lead to an asymmetrical feedback along the collaboration gradient, e.g., negative feedbacks with negative distances and neutral with positive. Similar asymmetry might occur along the conservation gradient if fast species are more tolerant of pathogen attack, meaning they do not suffer as much from their own pathogens as slow species do, or conversely if slow species are so well defended that they do not suffer from the pathogens cultured by the fast species. The significant effects of the midpoints that we found in our analysis might indicate that our simple assumptions are not supported and that the framework needs to be expanded. If these non-linear effects of each axis occur then we would also need to be careful when comparing results from different studies as the PCA axes produced (and therefore the midpoints along those axes) would not be comparable. In this case it may be possible to scale the axis found in a smaller study with a reduced species pool to the axis values found in more comprehensive analyses. However, more complete tests of the framework, with a wider set of species and distances, are needed to fully assess this.

For simplicity our framework assumes that pathogens and mutualists are entirely generalist and able to associate with species of any other strategy. However, many (fungal) pathogens are highly specialised and attack only one or a few related species (Gilbert and Webb 2007; Kembel and Mueller 2014; Rutten et al. 2021). In this case, we might see negative feedbacks occurring even when functionally very similar species are compared. To test this, we would need to control for phylogenetic distance between the species when designing and analysing PSF experiments. Including phylogenetic distance as an additional predictor in the model would be a straightforward way to achieve this. The collaboration gradient does show phylogenetic signal (Bergmann et al. 2020) so the correlation with phylogenetic distance would need to be assessed in each study. In addition, non-linear effects of phylogeny might occur if close relatives are much more likely to share pathogens or mutualists (Parker et al. 2015) and these non-linear phylogenetic distances would be less correlated with linear trait distances. Similarly, either balancing phylogenetic and functional distance in the species selection, or excluding comparisons between close relatives, would be a way to ensure that phylogenetic distance does not confound the distances along the collaboration and conservation gradients. The overall importance of specialist versus generalist microbes in affecting plant performance and plant soil feedbacks is not well known (Semchenko et al. 2022) but could be tested with such designs and analyses.

### Extensions of the framework

We have considered the role of pathogens and mutualists in driving plant soil feedbacks. However, additional mediators, such as saprotrophs, nitrogen fixing bacteria, nematodes, soil mesofauna and abiotic feedbacks, may play a role in affecting plant-soil feedbacks. For example, it has been suggested that decomposition rates depend on the dissimilarity of the home litter as compared to the litter of surrounding plants (Freschet et al. 2012), indicating that decomposers are likely to contribute to PSFs particularly when the distances along either axis increase. Moreover, it is well known that decomposer communities shift from fungal dominated in low resource environments to bacterial dominated in high resource environments (de Vries et al. 2012). If this shift accompanies a change in the root strategies of the plant species, i.e., from slow to fast, then we might expect different decomposer communities to associate with fast vs. slow species. In this case we could see reciprocal positive feedbacks caused by saprotrophs along the conservation gradient because plant species benefit from the more active decomposer community on their home soil (Ke et al. 2015; Veen et al. 2019). Such effects might not be seen in short term pot experiment but could be tested for by adding litter addition treatments to the plant soil feedback experiment or by conducting longer term experiments in more natural settings (De Long et al. 2019). As discussed above, legumes are likely to enjoy positive feedbacks and to behave differently from other species with high root N content (fast species). It might therefore be important to include a binary trait for nitrogen fixation in the analysis. In addition, many other bacterial species are mutualists and may reduce the attack by pathogens (e.g., Latz et al. 2012). It is not clear what root traits predict the abundance of these microbes but first studies have linked functional plant community characteristics to soil community responses (Leff et al. 2018; Mommer et al. 2018; Sweeney et al. 2021). Our framework could be used in combination with a characterisation of the soil communities cultured by plant species from each belowground strategy. We have assumed that the root traits mostly affect the abundance of pathogens and mutualists in soils but changes in diversity and composition of microbial communities are also likely to occur and these might further impact PSFs. Characterising the soil microbial community would allow us to assess this and we could test the relationship between the functional distance and beta-diversity between home and away soil community components (eg. pathogens or mutualists) along each gradient in root economics space. This might help to identify drivers of PSF in the soil community and functional trait effects on soil communities.

Our novel concept could also be extended to move away from pairwise species interactions and allow community level assessments of the four belowground strategies. Several studies have shown that soil biota can drive a positive relationship between biodiversity and plant productivity because soil pathogens reduce the performance of plant species in monoculture (Maron et al. 2011; Schnitzer et al. 2011; Mommer et al. 2018). Similar to a negative PSF_home/away_, plants benefit from dilution effects in mixtures because their pathogens do not accumulate to such an extent. Monocultures have also been shown to harbour specific pathogens not found in mixed communities (Mommer et al. 2018) and a progressive accumulation of pathogens in monoculture and of mutualists in mixtures has been suggested as a major driver of increasing biodiversity effects over time (Thakur et al. 2021). Applying our framework to biodiversity-functioning relationships, we would predict that mixing fast and slow should lead to the most effective dilution of pathogens because the fast species would benefit from growing next to resistant slow species, a mixture of fast species might not benefit from dilution because pathogens would still be abundant. Including outsourcer species in mixed communities would be likely to lead to highest productivity as they would increase mutualist abundance, however, a combination of DIY and outsourcer species could also be complementary if the mycorrhizae and the plants are better at accessing different nutrients or acquiring nutrients from different soil depths. Maximising diversity of root functional strategies in mixed communities might therefore lead to the highest productivity. It would be interesting to use root trait functional diversity as a predictor of productivity in biodiversity experiments to test this idea and to use root traits to explain differences in monoculture performance over time. In addition, functional traits can be linked to biodiversity effects on production (selection and complementarity; Loreau and Hector 2001; see eg. Jing et al. 2015; Cadotte 2017; Bakker et al. 2019; Cappelli et al. 2022). Linking root traits to biodiversity effects could be a promising way to test mechanisms by which root trait functional diversity might enhance plant productivity and ecosystem functioning.

## Conclusion

Plant soil feedbacks are the result of a complex interplay between plants, mutualists and antagonists and it remains difficult to predict when PSF should be strong. Here, we propose and test a novel concept that can help to predict the outcome of plant-soil feedbacks by linking plant belowground strategies to soil community components. We predict that pathogens and mutualists scale on independent, orthogonal axes of the recently described root economics space, leading to different soil communities for plants with different combinations of belowground strategies. As the variation along these gradients is continuous, we suggest that continuous measures of functional distance are needed to predict variation in plant-soil feedbacks, and we predict that there should be an interaction between the two distances. Our predictions would also suggest that soil communities could mediate competitive exclusion or coexistence between certain strategies and incorporating root traits into plant competition theory could be a promising future direction. The different effects of the various root strategies on soil microbes would also have consequences for understanding biodiversity functioning relations and might lead to different relationships when different combinations of root strategies are combined. Our first application of the frame work to data from two case studies provides some support for our predictions and the variables are able to explain almost half of the variation in plant-soil feedbacks between the species pairs. Our approach of predicting plant soil feedbacks using the functional similarity between the focal and the soil conditioning species, together with the position of the species pair along each gradient, could therefore be widely applied to analyse plant-soil feedbacks in multispecies experiments. We argue that this framework could lead to a more mechanistic understanding of the outcome of plant-soil feedbacks and that differences in root traits and the root strategies resulting from the root economics space may be key predictors of the strength and direction of plant-soil feedbacks.

## Supplementary Information

Below is the link to the electronic supplementary material.

Supplementary file1 (DOCX 57 KB)

## Declarations

This work was supported by the Swiss National Science Foundation (Award 31003A_160212).

Both authors contributed to the study conception and design. Material preparation, data collection and analysis were performed by GR. The first draft of the manuscript was written by GR and EA and both authors commented on previous versions of the manuscript. All authors read and approved the final manuscript.

The data and R scripts used for the current study are available in the Zenodo repository, [https://doi.org/10.5281/zenodo.7599998] and on GitHub (https://github.com/ggpmrutten/linkingRES-PSF).
