# Supplementary material for: Using root economics traits to predict biotic plant soil-feedbacks
Source: Plant Soil. 2023 Mar 2;485(1-2):71–89. doi: 10.1007/s11104-023-05948-1 (PMC10167139; doi:10.1007/s11104-023-05948-1)

Supplementary

**Table S1** Focal species (Home) used in the two case studies, with their location in the root economics spectrum (collaboration and conservation gradient) and their average plant soil feedback (PSF_live/sterile_).

| Species | collaboration gradient | conservation gradient | PSF_live/sterile_ |
| --- | --- | --- | --- |
| *Acer saccharum* | 11.90 | -0.88 | -0.09 |
| *Arrhenatherum elatius* | 1.73 | -16.24 | -1.59 |
| *Centaurea jacea* | -12.90 | 7.64 | -1.48 |
| *Dactylis glomerata* | 16.10 | -3.97 | -1.56 |
| *Festuca rubra* | 17.73 | -14.22 | -1.37 |
| *Fraxinus americana* | 0.99 | 0.85 | -0.62 |
| *Galium mollugo* | 18.31 | -1.93 | -1.21 |
| *Gleditsia triacanthos* | -0.18 | 12.89 | -0.11 |
| *Holcus lanatus* | 27.06 | 3.52 | -1.56 |
| *Leucanthemum vulgare* | 3.09 | -7.02 | -1.58 |
| *Medicago lupulina* | 7.21 | 35.58 | 0.59 |
| *Pinus strobus* | -18.02 | -0.93 | -0.05 |
| *Plantago lanceolata* | 13.19 | -4.98 | -2.10 |
| *Populus deltoides* | 31.26 | 2.60 | 0.19 |
| *Prunus serotina* | -2.40 | -9.04 | -1.17 |
| *Quercus rubra* | 8.79 | -19.13 | -0.22 |
| *Sassafras albidum* | -15.22 | 39.20 | -0.70 |
| *Trifolium pratense* | 4.55 | 15.79 | 0.16 |
| *Trifolium repens* | 10.37 | 27.10 | 0.47 |
| *Vicia cracca* | -35.16 | 18.34 | 0.07 |

**Table S2** Species pairs used in the two case studies, with their respective distance and mid points in the root economics spectrum and their measured PSFs_home/away_.


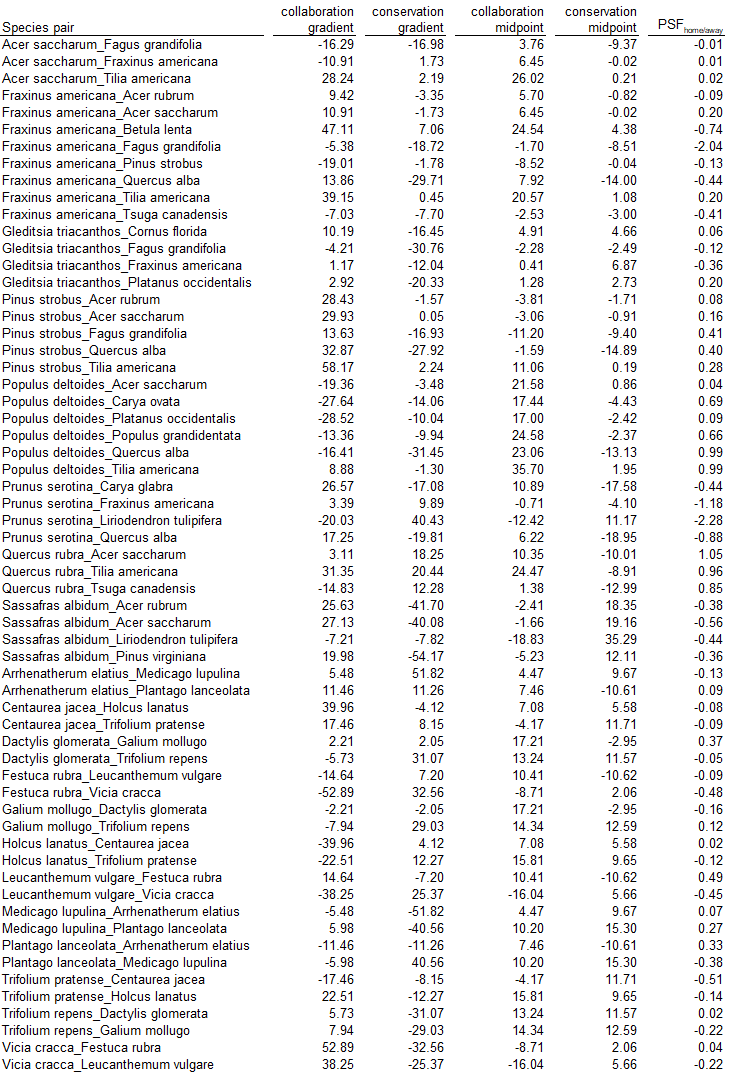

Supplement: Supplementary file 1 — Supplementary file1 (DOCX 57 KB) [file 11104_2023_5948_MOESM1_ESM.docx]
